# Critical phenomena of emergent magnetic monopoles in a chiral magnet

**DOI:** 10.1038/ncomms11622

**Published:** 2016-05-16

**Authors:** N. Kanazawa, Y. Nii, X. -X. Zhang, A. S. Mishchenko, G. De Filippis, F. Kagawa, Y. Iwasa, N. Nagaosa, Y. Tokura

**Affiliations:** 1 Department of Applied Physics, The University of Tokyo, Hongo 7-3-1, Bunkyo-ku, Tokyo 113-8656, Japan; 2 RIKEN Center for Emergent Matter Science (CEMS), Wako 351-0198, Japan; 3 SPIN-CNR and Dipartimento di Fisica, Università di Napoli Federico II, I-80126 Napoli, Italy

## Abstract

Second-order continuous phase transitions are characterized by symmetry breaking with order parameters. Topological orders of electrons, characterized by the topological index defined in momentum space, provide a distinct perspective for phase transitions, which are categorized as quantum phase transitions not being accompanied by symmetry breaking. However, there are still limited observations of counterparts in real space. Here we show a real-space topological phase transition in a chiral magnet MnGe, hosting a periodic array of hedgehog and antihedgehog topological spin singularities. This transition is driven by the pair annihilation of the hedgehogs and antihedgehogs acting as monopoles and antimonopoles of the emergent electromagnetic field. Observed anomalies in the magnetoresistivity and phonon softening are consistent with the theoretical prediction of critical phenomena associated with enhanced fluctuations of emergent field near the transition. This finding reveals a vital role of topology of the spins in strongly correlated systems.

## Abstract (web summary)

Phase transitions in topologically non-trivial systems are characterized by changes of topological invariants, rather than conventional order parameters. Here, the authors propose a real-space topological phase transition upon pair annihilation of emergent monopoles inherent in chiral magnet MnGe.

## Graphical abstract

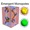

The search for phase transitions and critical phenomena beyond Landau's conventional scheme is one of the most fundamental issues in the physics of strongly correlated systems and topological quantum matter^1^. Symmetry breaking is the key concept of Landau, which is missing in the topological phase transitions. The topological order is characterized by an index such as the Chern number or *Z*_2_ number defined in the first Brillouin zone, the edge or surface gapless states and also the ground-state degeneracies for the non-trivial topological geometry of the sample^2^,^3^. The topological phase transition, that is, the change of the topological index defined in momentum space, usually requires the gap closing at some momenta and gives quantum critical phenomena with diverging correlation length. However, the topological phase transition defined in real space and its associated critical phenomena have never been explored in the context of the electronic states.

Topological spin orders in real space can be transcribed into the electronic state via the gauge field, that is, the emergent magnetic field^4^. A real-space geometric arrangement of spins affects charge transport by exerting an additional quantum phase on the itinerant electrons, called Berry phase^5^, which acts as the effective magnetic field. Among topological spin orders, hedgehogs and antihedgehogs play the roles of sources or sinks of emergent field, that is, emergent monopoles or antimonopoles^6^,^7^. These hedgehog spin structures have been indeed realized as topological defects such as Bloch points^8^,^9^,^10^,^11^ and singular points where magnetic skyrmions coalesce with or split from one another^12^. However, topological transitions associated with creation and annihilation of these hedgehogs are random events in terms of time and space; observation and identification of the consequent critical phenomena in the presence of emergent monopoles remain unexplored because of difficulty in controlling their behaviours.

Here we propose that the chiral magnet MnGe, where a periodic assembly of spin hedgehogs (monopoles) and antihedgehogs (antimonopoles) is realized as the magnetic ground state^13^,^14^,^15^, is an ideal system showing the topological phase transition of the real-space spin configuration and critical fluctuations acting on the conduction electrons and phonons. We have found that the Hall resistivity and magnetoresistivity (MR) in the magnetization processes show large deviations from conventional *M*- and *M*^2^-proportional profiles, which are accounted for by static and dynamic profiles of emergent fields, respectively. In particular, a prominent enhancement of positive MR and a large reduction in elastic constant are simultaneously observed on the pair annihilation of emergent monopoles, which highlights the role of the fluctuations of emergent field at the real-space topological phase transition.

## Results

### Hedgehog spin structures and emergent magnetic monopoles

**Figure 1:**
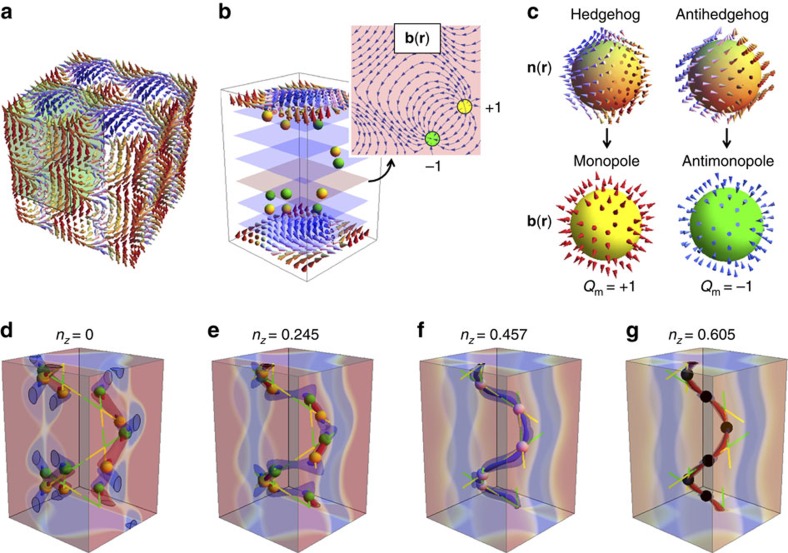
Spin configuration and corresponding emergent magnetic field with monopole solution in MnGe. (**a**) Spin orientation in a 2 × 2 × 2 magnetic unit cell. (**b**) Configuration of emergent monopoles (yellow dots) and antimonopoles (green dots) in a 1 × 1 × 1.5 magnetic unit cell corresponding to the green box in **a**, displayed with constant *z* planes (blue and pink planes). A stream of emergent field between a monopole and an antimonopole on the same *z* level is also exemplified (pink plane). (**c**) Hedgehog and antihedgehog spin arrangements **n**(**r**) realized in the spin structure of MnGe, which are regarded as quantized source (monopole; *Q*_m_=+1) and sink (antimonopole; *Q*_m_=−1) of emergent field **b**(**r**), respectively, acting on conduction electrons. (**d**–**g**) Monopoles' and antimonopoles' positions and their trajectories (yellow and green dots and lines) and distributions of emergent magnetic field under varying magnetic field applied along the *z* direction. Monopoles and antimonopoles collide at white dots (**f**) and pair annihilate at black dots (**g**). Three contours of emergent-field distribution, that is, regions with |*b*_*z*_|=10 and 2, and surfaces of green box of **a** with |*b*_*z*_|<0.1 are dyed according to strength and sign of *b*_*z*_—thicker colour for larger magnitude of *b*_*z*_, red and blue for positive and negative *b*_*z*_, respectively (**d**–**g**).

The magnetic structure in MnGe (shown schematically in Fig. 1a) has been observed by neutron diffraction^14^ and Lorentz transmission electron microscopy^15^ below the magnetic transition temperature *T*_N_≈170 K and is modelled by the superposition of three orthogonal helical structures induced by the Dzyaloshinskii–Moriya interaction (DMI)^16^,


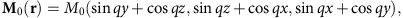


where *M*_0_ and *q* are the amplitude and the wavenumber of the helical moment, respectively; the periodic modulation length *λ*=2*π*/*q* is ranging from 3 nm (typically below 50 K) to 6 nm (near *T*_N_)^13^,^14^,^15^. In sharp contrast to the two-dimensional skyrmion crystal state^17^,^18^, there occur several points in the real space where **M**(**r**)=**0** (ref. 19). However, this large modulation in the magnitude of the spin moment is prohibited in the strong correlation limit, where the spin moment is saturated at low temperatures. Those genuine topological magnetic defects with zero magnetization, which are surrounded by saturated moments pointing in all directions, can be realized by the topological protection even though it is energetically costly, as discussed theoretically^8^,^9^ and demonstrated experimentally^10^,^11^. This means that 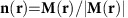 rather than **M**(**r**) is the more appropriate field that influences the conduction electrons in the strongly correlated systems. We regard that MnGe belongs to this case; in fact, its saturated magnetization *M*_s_=1.9 *μ*_B_ per f.u.^13^ is approximately five times enhanced as compared with the value of the related magnet MnSi^20^ with a larger one-electron bandwidth. Mathematically, **M**(**r**) belongs to *B*^3^ (inside the three-dimensional sphere), whereas **n**(**r**) belongs to *S*^2^ (surface of the three-dimensional sphere). The former is trivial, as all configuration of **M**(**r**) is smoothly connected to **M**(**r**)=**0**, whereas *S*^2^ is non-trivial as characterized by the Berry curvature 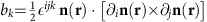, which corresponds to the solid angle subtended by **n**(**r**) and the emergent magnetic field acting on electrons in the same manner as the classical magnetic field does^4^. It can be seen that the points with **M**(**r**)=**0** correspond to the hedgehog or antihedgehog of **n**(**r**) with the effective magnetic charge 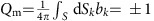 (*S* is the surface enclosing the singularity), namely the emergent magnetic monopole and antimonople (see Fig. 1c and also Supplementary Note 1)^6^,^7^,^8^,^9^,^10^,^11^,^12^,^21^,^22^,^23^. Therefore, the strong correlation limit produces the non-trivial topological classification of the spin configurations and the topological phase transition as well. It is noteworthy that this topological phase transition and associated critical phenomena are defined for the classical spin configuration **n**(**r**) and its thermal fluctuations at finite temperatures. It is also worth noting that the topology is defined for the continuous function **n**(**r**) and becomes less rigorous for **n**_*i*_ defined on the atomic lattice. Therefore, the experimental signature should be detected as anomalies instead of the singularities of physical properties.

Figure 1 shows the exchange field **n**(**r**) (Fig. 1c) and the corresponding emergent field **b**(**r**) (Fig. 1c–g). Zero points of **M**(**r**) dress hedgehog-type topological spin textures around them, resulting in the singular points of **n**(**r**), and behave as emergent monopoles (Fig. 1c), which are dotted in the magnetic structure exemplified in Fig. 1b. A stream plot of emergent magnetic field of a monopole–antimonopole pair on the identical *z*-plane clearly demonstrates that the emergent monopole and antimonopole act as its source and sink, respectively (inset of Fig. 1b).

**Figure 2:**
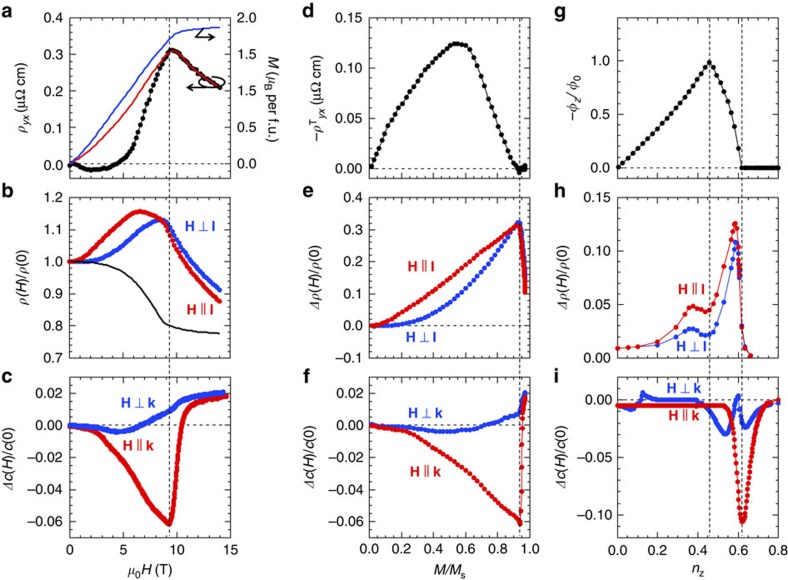
Experimental and theoretical results on unusual magneto-transport and elastic properties originating from emergent monopoles. (**a**–**c**) Experimental results on magnetic-field dependences of magnetization (blue line), expected usual Hall resistivity (red line) due to normal and anomalous Hall effects and observed Hall resistivity (dots) (**a**); expected usual MR (black line) and observed longitudinal and transverse MRs (**b**) change in elastic constants with propagation directions parallel and perpendicular to the magnetic field (**c**) at 30 K. Experimental estimates of topological Hall resistivity (**d**) positive contributions to MRs (**e**), which deviate from the usual MR profile (black curve in **b**), and relative elastic constants (**f**) at 30 K as functions of normalized magnetization *M*/*M*_s_, *M*_s_ being the low-temperature and high-field (for example, 2 K and 14 T) saturated value of *M*. (**g**–**i**) Theoretical calculations on static emergent magnetic flux −*φ*_*z*_ in a unit of *φ*_0_=*h*/*e* (**g**) longitudinal and transverse MRs originating from emergent field (**h**) elastic constants (**i**) as functions of the polarization factor *n*_*z*_.

Now we turn to the magnetization process. The topological spin texture under the magnetic field along the *z* direction is approximately expressed by the formula **M**(**r**)=**M**_0_(**r**)+(0,0,*m*_*z*_); *m*_*z*_ is introduced so as to concisely represent a uniform magnetization induced by an external magnetic field along the *z* axis. An external magnetic field does not spoil the topological singularity up to some critical field, while shifting the positions of monopoles and antimonopoles along yellow and green trajectories, respectively, as shown in Fig. 1d–g: their collision occurs at *n*_*z*_=0.457 and the pair annihilation at *n*_*z*_=0.605, where we introduce the polarization factor 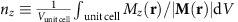 to compare the transport data shown in Fig. 2. As it can be seen from contour mappings of the emergent field in Fig. 1d–g, the regions of strong emergent field, which are constricted in small volumes and connect monopoles and antimonopoles, vary significantly according to monopoles' positions. At finite temperatures, the spin structure and the corresponding monopoles' positions can thermally fluctuate, thereby resulting in fluctuating emergent magnetic fields. Such fluctuations are expected to be greatly enhanced, in particular near the second-order phase transition associated with the monopole–antimonopole pair annihilation. As a hallmark of unconventional electromagnetic responses exemplifying such critical phenomena of the topological phase transition, below we discuss an unusually large, positive MR and elastic softening.

### Critical phenomena in transport and elastic properties

We first show anomalous magneto-transport and elastic properties observed at 30 K, which we attribute to consequences of static and dynamic properties of emergent fields. Figure 2a–c shows magnetic-field dependence of Hall resistivity *ρ*_*yx*_, MR *ρ*(*H*)/*ρ*(0) and relative elastic constant Δ*c*(*H*)/*c*(0), respectively. In Fig. 2a, we reproduce the topological Hall effect in MnGe as reported in refs. 13 and 14. In addition to the normal and anomalous Hall effects, that is, conventional features appearing as *H*- and *M*-linear transverse voltages in metallic magnets^24^, the topological Hall effect due to the real-space emergent magnetic field is observed as a hallmark of formation of a non-coplanar spin structure^25^,^26^. A clear deviation from the expected conventional Hall response *ρ*_*yx*_=*R*_0_*H*+*S*_*H*_*ρ*^2^*M* (red curve in Fig. 2a) represents the averaged static emergent field^13^. Here we note that transverse thermoelectric effect due to the emergent field (topological Nernst effect) is also observed in MnGe, which further supports the existence of emergent magnetic fields^27^.

We show in Fig. 2b a large, positive MR in the course of magnetization alignment by magnetic field; this is in stark contrast to cases of other isostructural helimagnets of MnSi^28^ and Mn_1−*x*_Fe_*x*_Ge without emergent monopoles (see Supplementary Note 2 and Supplementary Fig. 1), which show the conventional negative MR where spin-dependent scattering of conduction electrons reduces in the magnetization process, resulting simply in decrease of the resistance, that is, negative MR^29^. Both longitudinal (**H**||**I**, red curve in Fig. 2b) and transverse (**H**⊥**I**, blue curve in Fig. 2b) MRs largely depart from the estimated magnetic-field dependence of the conventional negative MR (black curve in Fig. 2b, see Supplementary Fig. 2 and Supplementary Note 3 for the experimental procedure to estimate it) and reach the maximal value near the magnetic phase boundary to the induced ferromagnetic state.

Next, we present in Fig. 2c the ultrasonic responses with sound wave propagating parallel (**H**||**k**; **k** being the propagation vector) or orthogonal (**H**⊥**k**) to the gradually increasing magnetic field. A huge elastic softening around the magnetic transition, that is, the topological transition, as well as a broad tail below the critical field are discerned in the parallel case, whereas the orthogonal case shows a moderate magnetic-field dependence with a multi-peak-and-trench fine structure (see Supplementary Fig. 3 and Supplementary Note 4 for clarity of the fine structure). This is also in stark contrast to the known case of the magnetic skyrmions in MnSi^30^, where elastic responses occur in a stepwise manner in going from conical phase to skyrmion phase but show minimal magnetic-field dependence within each phase. In the present case of MnGe, the elastic response is an order of magnitude larger than the conventional case (for example, in MnSi), showing up as the critical elastic softening around the topological phase transition (for **H**||**k**) and also as the fine structure in the magnetization process (for **H**⊥**k**). It is noteworthy that there is no distinct or discontinuous anomaly in the corresponding magnetization curve (see Fig. 2a).

### Comparison with theoretical predictions

The above experimental results are consistent with theoretical predictions of unique features associated with the monopole dynamics in MnGe. To clarify the role of emergent magnetic field, we extract the unusual contributions and compare them with theoretical calculations taking account of emergent-field effect and its spatial and temporal variations via spin-wave excitations (see Methods for detail). Figure 2d–f respectively presents the deviations from conventional Hall resistivity and negative MR, and the elastic constants as functions of normalized magnetization *M*/*M*_s_, where *M*_s_ is a saturated magnetization defined as the value at low-temperature and high-magnetic field, for example, at *T*=2 K and *μ*_0_*H*=14 T. Theoretical counterparts are shown in Fig. 2g–i as functions of *n*_*z*_, from which we find the following characteristics. Variation of monopoles' positions against magnetic-field change (Fig. 1d–g) results in continuous change in total emergent magnetic flux 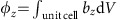. As shown in Fig. 2g, *φ*_*z*_=0 at zero magnetic field (*n*_*z*_=0) due to cancellation between positive and negative contributions, the maximum absolute value *φ*_*z*_=−*φ*_0_ at the colliding point (*n*_*z*_=0.457) of monopoles and antimonopoles, and *φ*_*z*_=0 at the pair annihilation point (*n*_*z*_=0.605) (see also Supplementary Fig. 4 and Supplementary Note 5), reproducing the experimental observation of the topological Hall resistivity as a function of *M*/*M*_s_ shown in Fig. 2d. (The disagreement between the corresponding experimental *M*/*M*_s_ and theoretical *n*_*z*_ values arises from the crude assumption that **M**(**r**)=**M**_0_(**r**)+(0, 0, *m*_*z*_), to simplify the magnetic-field effect on the deformation of magnetic structure.) This continuity also ensures that the topological phase transition due to the pair annihilation is of the second order; indeed, no hysteretic or discontinuous behaviour is experimentally observed^13^. The large, positive MR reaching the maximum around the magnetic phase boundary is ascribed to the fluctuations of emergent magnetic field that is critically enhanced around the monopole–antimonopole annihilation point, as represented in Fig. 2h. Although in general various mechanisms can simultaneously contribute to MR (see Supplementary Note 2 and Supplementary Fig. 1 for investigation of other possibilities), we found that the MR profile in MnGe can be well accounted for by considering fluctuations of emergent magnetic field, which are evaluated by calculating the correlation functions 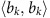 (*k*=*x*, *y*, *z*). The calculated 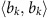 exhibits an anisotropy 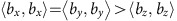 and gets larger towards the upsurge at the phase transition (see Supplementary Fig. 5 and Supplementary Note 6), thus reproducing the experimental observations of MR (Fig. 2e). Spin-wave excitations in the monopole–antimonopole crystal, which are composed of three modes (linear, quadratic and gapped dispersions as functions of momentum), have much lower energies than the temperature and play a key role in a microscopic process of the inelastic scattering of conduction electrons by monopole fluctuations (see Methods). In line with this model, modification of magnetic interactions due to the sound-wave-induced strain is responsible for the observed elastic anomalies (Fig. 2c). In reality, mixing up the three-mode magnon spectrum and the acoustic phonon spectrum results in a dramatic elastic softening around the phase transition for the parallel (**H**||**k**) case as well as a multi-peak-and-trench elastic response for the orthogonal (**H**⊥**k**) case, as shown Fig. 2i. (See Methods, Supplementary Fig. 3 and Supplementary Note 4 as well.)

We thus find good agreements between the experimentally observed anomalies and the theoretical predictions at these extremal–*φ*_*z*_ and zero–*φ*_*z*_ (pair annihilation) points. However, the calculated dip in MR around *n*_*z*_=0.457 (indicated with a vertical dashed line in Fig. 2h), which is ascribed to the suppression of the fluctuations of the emergent magnetic field at the extremum of *φ*_*z*_, is only barely discernible in the experimental results at a restricted temperature region (see Supplementary Fig. 2). Although the fluctuation effect appears just around the phase transition (*n*_*z*_=0.605) in the calculations based on the random phase approximation, the elastic softening is experimentally observed in a broader magnetic-field range as the manifestation of extended fluctuations of monopole and antimonopole. Such robust fluctuations beyond the mean field picture may obscure the MR dip structure expected to be seen at the intermediate magnetic field with the extremum of *φ*_*z*_ and may also explain the broad tail of the elastic-constant anomaly below the critical field *H*_c_ (Fig. 2f).

**Figure 3:**
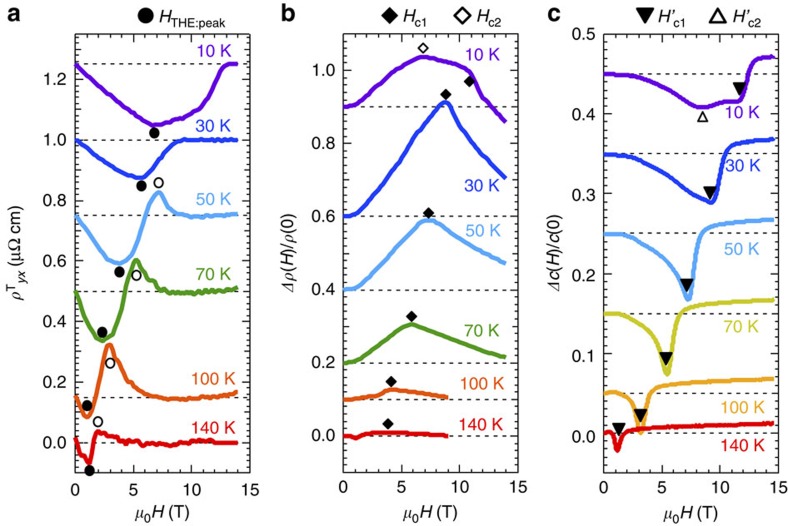
Experimental results on temperature development of transport and elastic properties. Magnetic-field dependences of topological Hall resistivity (**a**), longitudinal MR (**b**) and change in elastic constant with propagation directions parallel to the magnetic field (**c**). Characteristic magnetic fields are marked as closed circles (negative peaks of topological Hall resistivity representing the maximal emergent magnetic field |*b*_*z*_|; *H*_THE:peak_), open circles (positive peaks of topological Hall resistivity near the phase boundary), closed diamonds (peak in MR originating from fluctuations around phase boundary; *H*_c1_), open diamond (a peak in MR around the extremal-*φ*_*z*_ point; *H*_c2_), closed triangles (negative peaks in elastic constant originating from fluctuations around the phase boundary; 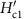) and open triangle (a kink in elastic constant around the extremal-*φ*_*z*_ point; 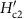).

**Figure 4:**
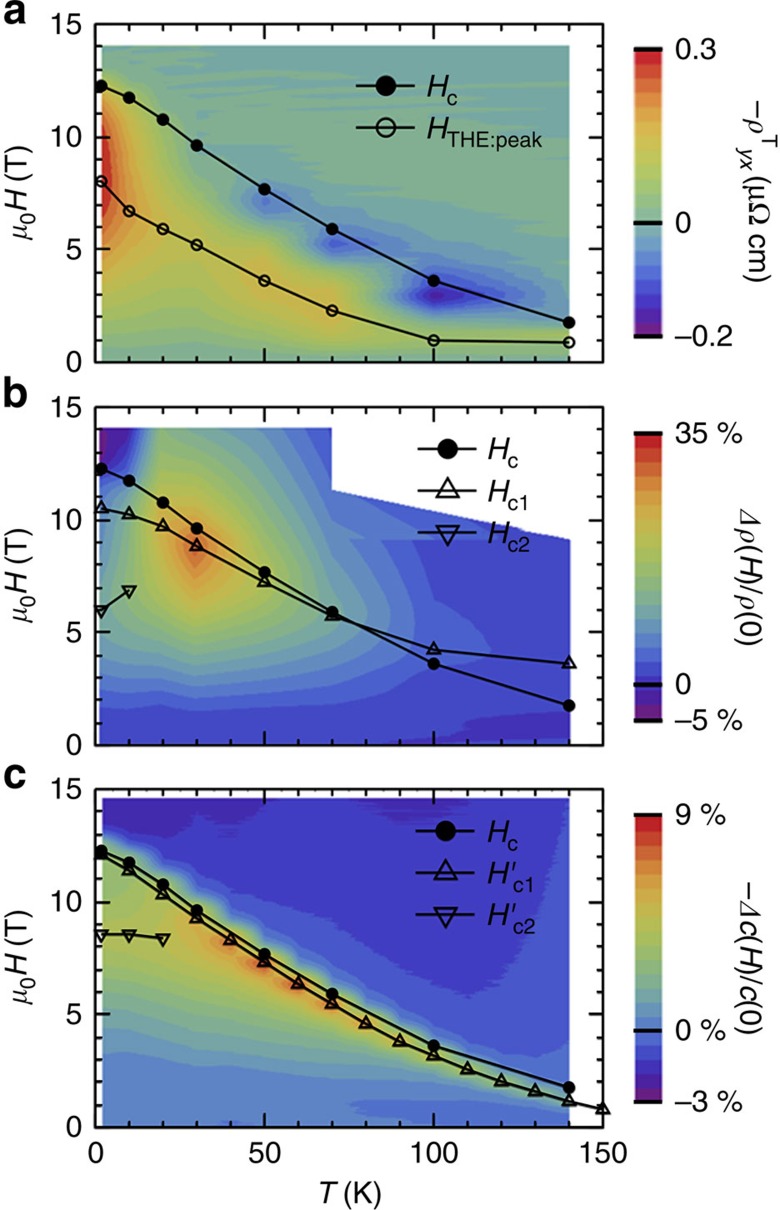
Static and dynamic properties of emergent monopoles highlighted by contour mappings of unusual magneto-transport and elastic properties. Contour mappings of topological Hall resistivity (**a**), positive contribution to longitudinal MR (**b**) and elastic constant with propagation directions parallel to the magnetic field (**c**) in *T*–*H* plane. The critical fields (*H*_c_) are defined as inflection points in *M*–*H* curves. The magnetic field where the topological Hall resistivity shows its extremum (*H*_THE:peak_) and other characteristic magnetic fields (*H*_c1_, *H*_c2_, 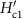 and 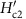) are also indicated in the corresponding panels.

Figure 3 shows the data sets of the monopole-related transport properties at various temperatures. Although the characteristic properties listed above are observed in a broad range of temperature where the spin hedgehog–antihedgehog crystal is formed^13^,^14^,^15^, the following features are further identified: peak structures in topological Hall resistivity show up at the pair annihilation points above 50 K (open circles in Fig. 3a) and also additional kinks in MR and elastic constant appear around the extremum point of topological Hall effect, that is, the extremal–*φ*_*z*_ point (open diamond and open up-triangle in Fig. 3b,c, respectively). However, those that remain unexplained may originate from emergent monopoles as well, given their close ties to the critical magnetic-field points; the corresponding magnetic fields *H*_c2_ and 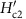 for the additional anomalies in MR and elastic constant are later presented in Fig. 4b,c.

With this reservation, we can separately assess these characteristic features from static and dynamic responses of emergent magnetic monopoles. We show in Fig. 4 contour mappings of the topological Hall resistivity (a), the positive MR (b) and the elastic constant (c) in the *T*–*H* plane. In Fig. 4a, the strong signal of negative topological Hall effect 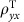, that is, emergent field strength *φ*_*z*_, shows up at intermediate external fields as characterized by the magnetic field with the negative peak of 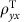 (*H*_THE:peak_) and continuously disappears at the phase boundary. In contrast, the strong signals of positive MR and elastic softening, which we assign to the outcomes of *φ*_*z*_ fluctuations, appear around the critical field *H*_c_ for the monopole–antimonopole pair annihilation as enhanced in an intermediate temperature region, for example, 20–70 K below *T*_N_. Coincidences of the critical field *H*_c_ and characteristic fields with peak signals of MR and elastic softening (*H*_c1_ and 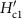) in the wide temperature range further support that the observed anomalies are the critical phenomena associated with continuous phase transition on the monopoles' pair annihilation.

The observed critical behaviours in MR and elastic property are well accounted for by the theoretical models, considering fluctuations of emergent monopoles, and hence represent the topological phase transition associated with the pair annihilation of the real-space hedgehog spin singularities in MnGe. The topology of spin structure, guaranteed by the three-mode helical magnetic ordering, enables the topological phase transition to survive even at finite temperatures, which can realize versatile unique phenomena based on the dynamics of emergent magnetic monopoles.

## Methods

### Sample preparation

Polycrystalline MnGe samples were synthesized with a cubic-anvil-type high-pressure apparatus. Alloys with stoichiometric quantities of the constituents were prepared by arc melting under an argon atmosphere, followed by heat treatment at 1,073 K under a high pressure of 4.0 GPa for 1 h. The material was confirmed to be single phase of B20-type chiral cubic structure at room temperature by powder X-ray analyses.

### Transport measurements

MR and Hall resistivity were measured by using AC-transport option in Physical Property Measurement System.

### Elasticity measurements

A phase comparison method^31^ was used for measurements of relative change in elastic constant. The sample has a cuboid shape with dimensions of 3.8 × 1.8 × 1.8 mm^3^. Two 36° *Y*-cut LiNbO_3_ piezoelectric transducers were attached on polished parallel surfaces for generation and detection of ultrasound. Longitudinal ultrasound having a frequency of 18 MHz was injected along the longest direction under the magnetic field parallel or perpendicular to its propagation direction **k**.

### Spin-wave theory

For a spin helix 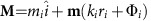 propagating along the *i*-direction (*i*=*x*, *y*, *z*), we define spin-wave fields *φ*_*i*_ and *δm*_*i*_ as the fluctuation part of the phase Φ_*i*_ and the uniform magnetization *m*_*i*_, respectively. The imaginary-time spin-wave Lagrangian density for hedgehog–antihedgehog lattice takes the form,


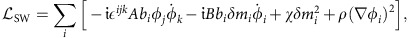


where 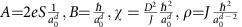, *e* is electric charge and *S* the spin magnitude, *a*_0_ the lattice constant and *d*=3 the spatial dimensions. The emergent magnetic field *b*_*i*_ are spatially averaged within a magnetic cell, as we are interested in the long-wavelength behaviour. The exchange interaction (EXI) and DMI stabilizing the hedgehog–antihedgehog lattice herein enter via the their strengths *J* and *D*, respectively. The first two terms originate from the spin Berry phase and the last two manifest the rigidity gained after symmetry breaking on the formation of hedgehog–antihedgehog lattice. The key feature consists in a three-mode magnon spectrum (linear, quadratic in momentum (*k*) and gapped, respectively) of this theory (see also Supplementary Fig. 3 and Supplementary Note 4).

### Resistivity calculation

Employing an adiabatic approximation for the real-space Berry phases produced by the spin moments of hedgehogs and meanwhile felt by itinerant electrons, one can derive an effective Hamiltonian for the electrons 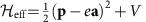 from a double-exchange model of Hund's rule coupling between electrons and localized spin moments. The interaction with spin-wave fluctuations enters via the spatial and temporal dependence of the emergent vector potential **a** and a potential *V* on the spin configurations. We take these fluctuations as first-order deviations away from the ground state, adopt the memory function method^32^ to calculate the finite temperature correlation function 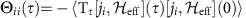 (*i*=*x*, *y*, *z*) and extract the dc resistivity by a numerical analytical continuation method^33^. This approach takes care of the gauge choice problem posed by the emergent monopolar field **b**=**∇** × **a** in a concise manner. Moreover, equal-time finite temperature correlation functions of the emergent magnetic fields can be calculated as well, so as to compare the fluctuations as stated in the main text.

### Magnetoelastic interaction

Two types of magnetoelastic coupling originate from the expansion of the EXI/DMI strength *J*/*D*:


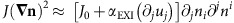


and


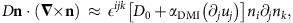


wherein we retain up to the first-order coupling coefficient *α*_EXI/DMI_ and incorporate the longitudinal acoustic phonon degrees of freedom, the displacement field *u*_*j*_. We then introduce spin-wave fields denoted collectively by a six-component field *ϕ*_*μ*_=(**φ**, δ**m**) and derive the interaction Lagrangian density of the form,


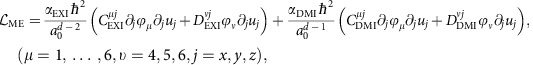


wherein *C*_EXI/DMI_ and *D*_EXI/DMI_ are matrices of some form factors embedding simultaneously the information of the ground-state spin configuration and the relevant form of magnetoelastic coupling derived from EXI/DMI. Together with 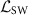 and a standard longitudinal phonon theory, by integrating out the spin-wave fields, we can attain an effective phonon theory that well describes the new phonon excitations under the hybridization with magnon modes. A new renormalized *k*-linear phonon mode as identified by examining the spectral function takes direct charge of the elastic responses.

(Detailed calculation results of the MR and the ultrasonic responses for more general cases with the use of a broader range of parameter values can be found in the papers in preparation by Zhang *et al*., and by Zhang and Nagaosa, respectively.)

## Additional information

**How to cite this article:** Kanazawa, N. *et al*. Critical phenomena of emergent magnetic monopoles in a chiral magnet. *Nat. Commun.* 7:11622 doi: 10.1038/ncomms11622 (2016).

## Supplementary Material

Supplementary Information Supplementary Figures 1-5, Supplementary Notes 1-6 and Supplementary References
