# Supplementary material for: Critical phenomena of emergent magnetic monopoles in a chiral magnet
Source: Nat Commun. 2016 May 16;7:11622. doi: 10.1038/ncomms11622 (PMC4873648; doi:10.1038/ncomms11622)
Supplement: Supplementary Information — Supplementary Figures 1-5, Supplementary Notes 1-6 and Supplementary References [file ncomms11622-s1.pdf]

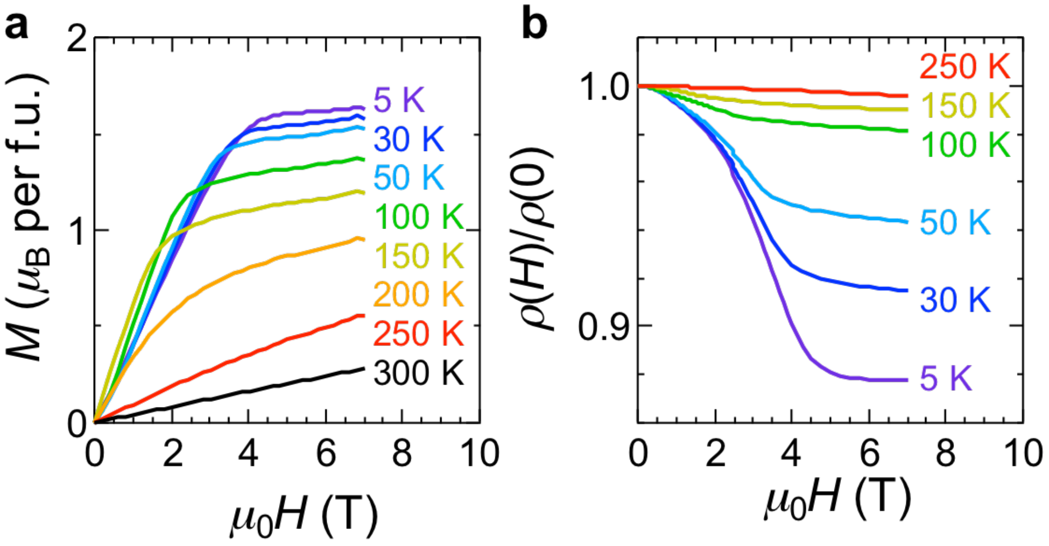

Supplementary Figure 1 │Magnetization and magnetoresistivity in $Mn_{0.7}Fe_{0.3}Ge$. (a,b) Magnetic field dependence of magnetization (**a**) and magnetoresistivity (**b**) at various temperatures.

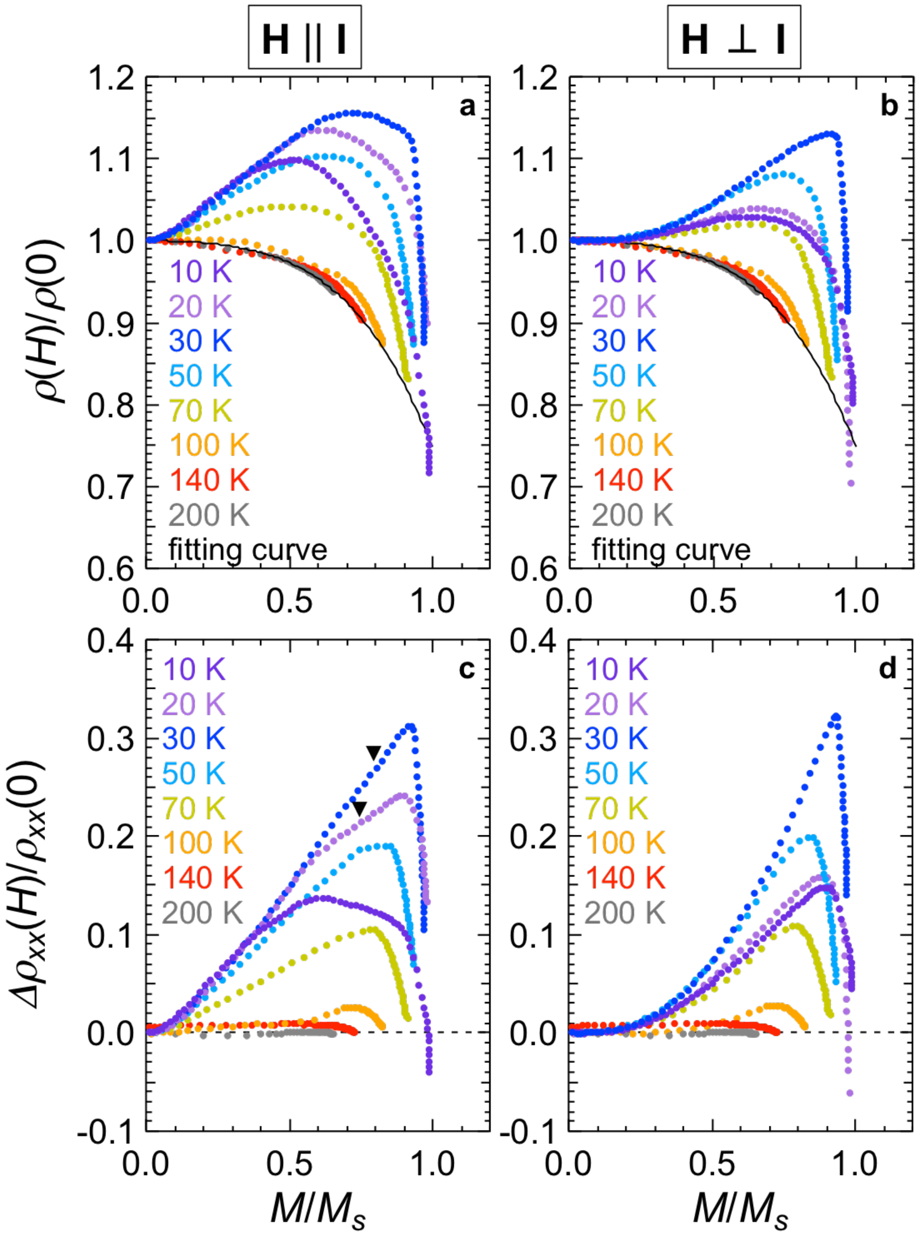

Supplementary Figure 2 │Longitudinal and transverse magnetoresistivity at various temperatures compared with estimated usual negative magnetoresistivity. (**a**,**b**) Longitudinal (**a**) and transverse (**b**) magnetoresistivity as functions of normalized magnetization M/M_s. Black curves indicate the fitting curve to magnetorestivity at 200 K. (**c**,**d**) Unusual contributions to longitudinal (**c**) and transverse (**d**) magnetoresistivity. Small dip structures observed at 20 K and 30 K are indicated by triangles.

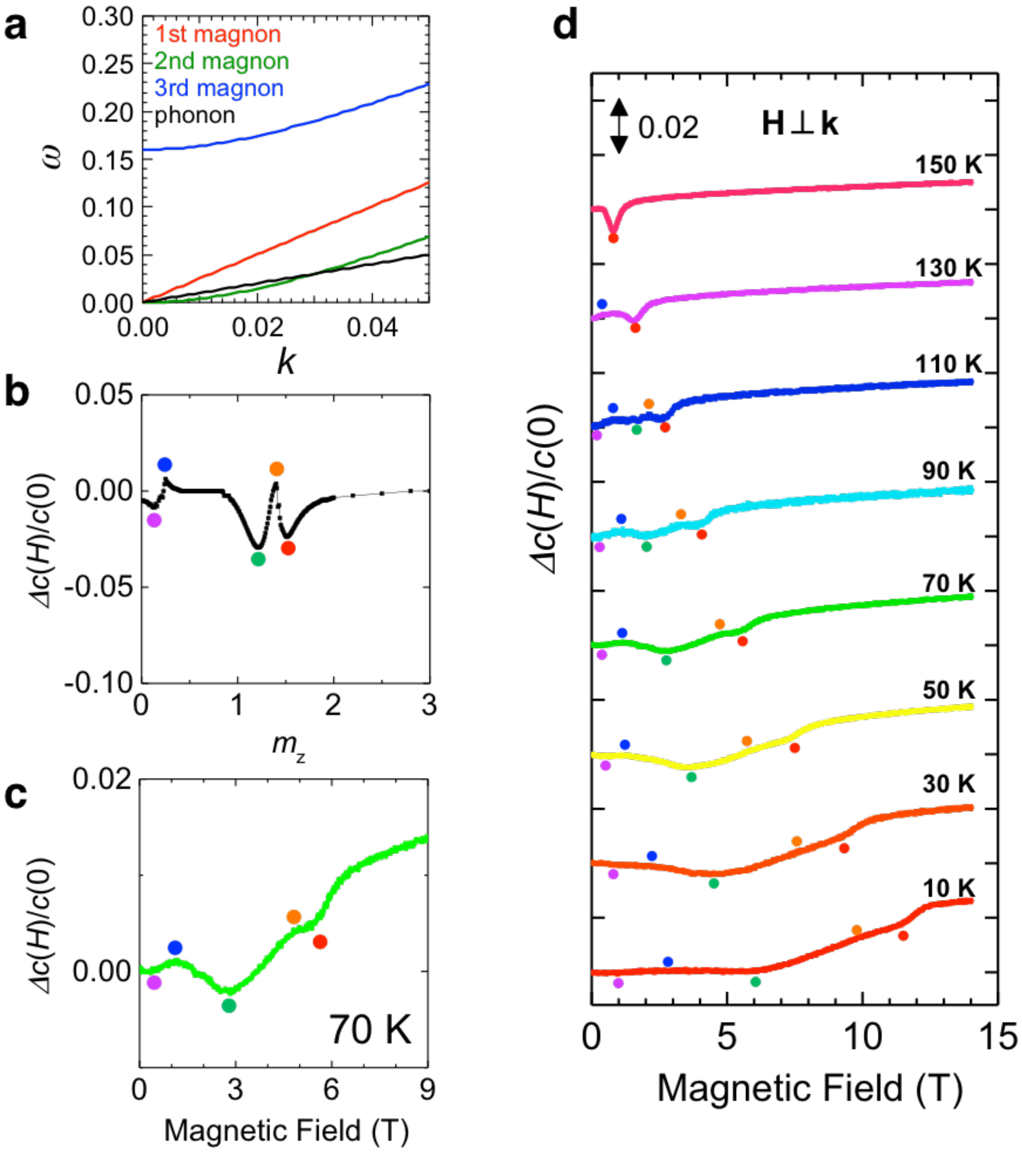

Supplementary Figure 3 | Magnon and phonon spectra, and theoretical and experimental elastic constant with sound propagation direction perpendicular to magnetic field. (**a**) Three-mode magnon spectrum and linear acoustic phonon spectrum. (**b**,**c**) Typical theoretical and experimental results on the elastic constant with $\mathbf{H} \perp \mathbf{k}$. Small multiple peaks and dips (indicated by colour dots) are observed in the both results, which show good correspondence with each other. (**d**) Temperature development of elastic constant (experimental) with $\mathbf{H} \perp \mathbf{k}$.

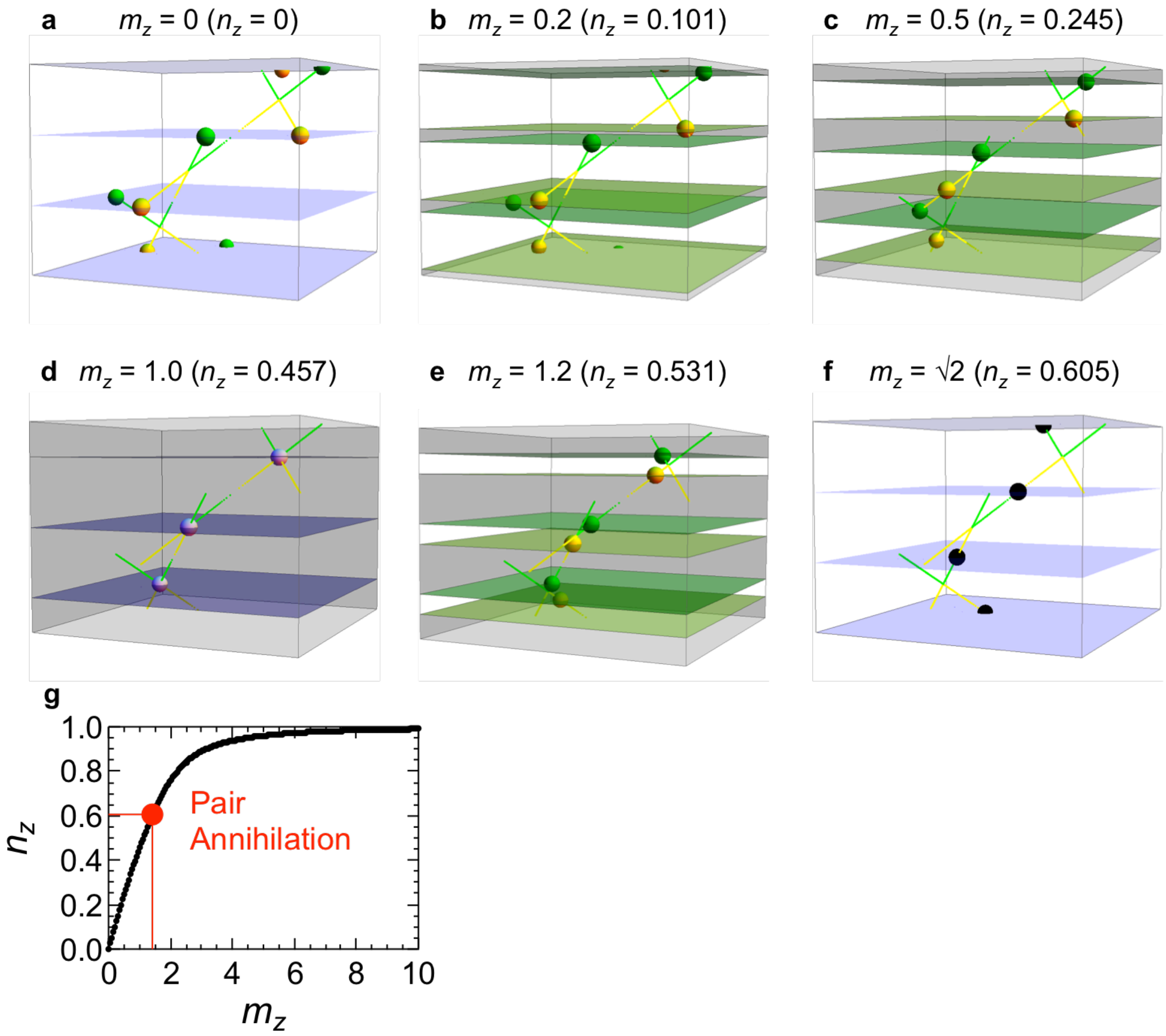

Supplementary Figure 4 │Magnetic-field dependence of monopoles' positions and regions with finite skyrmion number ($N_{\text{sk}} = -1$). (**a-f**) Monopoles (yellow dots) and antimonopoles (green dots) move along their trajectories (yellow and green lines) with changing magnetic field (induced magnetization m_z and polarization factor n_z). They collide at white dots (**d**) and annihilate at black dots (**f**). The gray shaded boxes indicate the regions with finite skyrmion number ($N_{\text{sk}} = -1$). (**g**) Relation between the magnetic-field-induced uniform magnetization m_z and the polarization factor n_z. The pair annihilation of monopoles and antimonopoles occurs at $m_z = \sqrt{2}$, $n_z = 0.605$ (red dot).

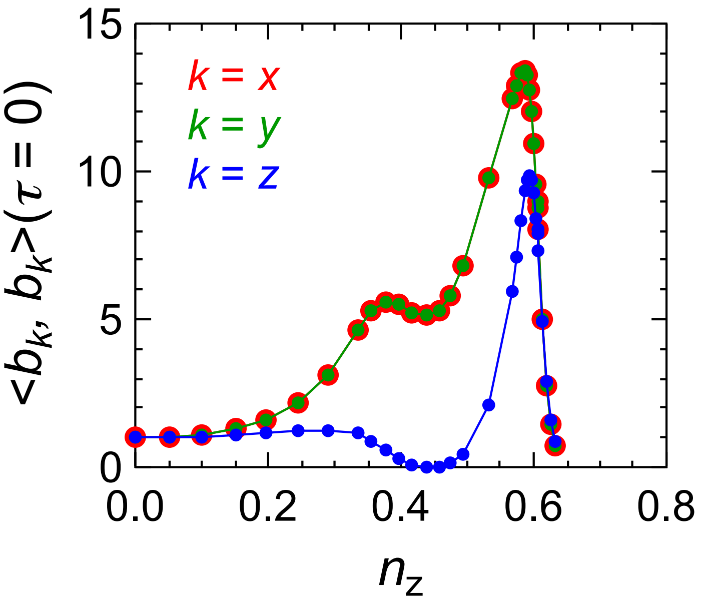

Supplementary Figure 5 │Magnetic-field dependence of emergent field fluctuation. The correlation functions $\langle b_i, b_i \rangle$ $(i = x, y, z)$ are plotted as functions of the polarization factor n_z.

Supplementary Note 1 │Monopole-type emergent fields from hedgehog spin structures in MnGe.

We describe in detail the magnetic structure model hosting the hedgehog spin structures and their corresponding emergent magnetic fields.

The model for the spin structure in MnGe is the superposition of three helical structures with their modulation directions being orthogonal to one another, as described in the main text:

$$\mathbf{M}(\mathbf{r}) = (\cos z, \sin z, m_z) + (\sin y, 0, \cos y) + (0, \cos x, \sin x)$$
$$= (\sin y + \cos z, \sin z + \cos x, \cos y + \sin x + m_z)$$

Hereafter, we consider the case of $m_z = 0$. There are eight singular points with $\mathbf{M}(\mathbf{r}) = \mathbf{0}$ in the magnetic unit cell, which correspond to hedgehogs and antihedgehogs of the unit vector $\mathbf{n}(\mathbf{r}) = \mathbf{M}(\mathbf{r})/|\mathbf{M}(\mathbf{r})|$ as follows[1]:

$$\text{Hedeghogs} = \left(\frac{\pi}{4},\frac{3\pi}{4},\frac{-3\pi}{4}\right),\left(\frac{-\pi}{4},\frac{-\pi}{4},\frac{-\pi}{4}\right),\left(\frac{3\pi}{4},\frac{-3\pi}{4},\frac{\pi}{4}\right),\left(\frac{-3\pi}{4},\frac{\pi}{4},\frac{3\pi}{4}\right),$$
$$\text{Antihedgehogs} = \left(\frac{-\pi}{4},\frac{\pi}{4},\frac{-3\pi}{4}\right),\left(\frac{\pi}{4},\frac{-3\pi}{4},\frac{-\pi}{4}\right),\left(\frac{-3\pi}{4},\frac{-\pi}{4},\frac{\pi}{4}\right),\left(\frac{3\pi}{4},\frac{3\pi}{4},\frac{3\pi}{4}\right).$$

Now we demonstrate that the emergent fields around the singular points indeed show the quantized monopole solution, i.e., $Q_m = \frac{1}{4\pi}\int_S \mathbf{b}\cdot d\mathbf{S} = \pm 1$, by holding up the case of the singular point $\left(\frac{-\pi}{4},\frac{-\pi}{4},\frac{-\pi}{4}\right)$ as an example. The corresponding emergent magnetic field can be calculated by substituting the magnetic model in the definition of Berry curvature:

$$\mathbf{b} = \frac{1}{|\mathbf{M}|^3}\left[\mathbf{M}\cdot(\partial_y\mathbf{M}\times\partial_z\mathbf{M}), \mathbf{M}\cdot(\partial_z\mathbf{M}\times\partial_x\mathbf{M}), \mathbf{M}\cdot(\partial_x\mathbf{M}\times\partial_y\mathbf{M})\right]$$
$$= \frac{1}{|\mathbf{M}|^3}\begin{pmatrix}\sin y + \cos z + \sin x\cos y\cos z + \cos x\sin y\sin z\\ \cos x + \sin z + \sin x\cos y\sin z + \cos x\sin y\cos z\\ \sin x + \cos y + \sin x\sin y\cos z + \cos x\cos y\sin z\end{pmatrix}.$$

Around the singular point $\left(\frac{-\pi}{4}+\delta x, \frac{-\pi}{4}+\delta y, \frac{-\pi}{4}+\delta z\right)$, the emergent field is expressed by the first order of $\delta\mathbf{r}=(\delta x, \delta y, \delta z)$ as $\mathbf{b}=\frac{1}{\sqrt{2}|\mathbf{M}|^3}\delta\mathbf{r}$. Then, we change the origin to be the singular point $\left(\frac{-\pi}{4}, \frac{-\pi}{4}, \frac{-\pi}{4}\right)$ and transform the variable from (x, y, z) to (ξ, η, ζ) via unitary matrices $U=\begin{pmatrix} 1/\sqrt{3} & 1/\sqrt{3} & 1/\sqrt{3} \\ 1/\sqrt{2} & -1/\sqrt{2} & 0 \\ 1/\sqrt{6} & 1/\sqrt{6} & -2/\sqrt{6} \end{pmatrix}$ and $U^{\dagger}=\begin{pmatrix} 1/\sqrt{3} & 1/\sqrt{2} & 1/\sqrt{6} \\ 1/\sqrt{3} & -1/\sqrt{2} & 1/\sqrt{6} \\ 1/\sqrt{3} & 0 & -2/\sqrt{6} \end{pmatrix}$, where $(\xi, \eta, \zeta)=U(x, y, z)$ and $(x, y, z)=U^{\dagger}(\xi, \eta, \zeta)$. Under the polar coordinate expression, every particular item necessary for the calculation of $\int_S \mathbf{b}\cdot d\mathbf{S}$ is described as follows:

$$\mathbf{r}=\begin{pmatrix} x \\ y \\ z \end{pmatrix}=U^{\dagger}\begin{pmatrix} \frac{R}{\sqrt{2}}\cos\theta \\ \sqrt{2}R\sin\theta\cos\phi \\ \sqrt{2}R\sin\theta\sin\phi \end{pmatrix},$$

$$d\mathbf{S}=\left(\partial_\theta\mathbf{r}\times\partial_\phi\mathbf{r}\right)d\theta d\phi=R^2\sin\theta\begin{pmatrix} 2\cos\theta \\ \sin\theta\cos\phi \\ \sin\theta\sin\phi \end{pmatrix}d\theta d\phi,$$

$$|\mathbf{M}|^2=2\xi^2+\frac{1}{2}(\eta^2+\zeta^2)=R^2.$$

Thus, the magnetic charge of the emergent field is

$$\frac{1}{4\pi}\int\mathbf{b}\cdot d\mathbf{S}=\frac{1}{4\pi}\int\frac{R}{\sqrt{2}R^3}\begin{pmatrix} \frac{\cos\theta}{\sqrt{2}} \\ \sqrt{2}R\sin\theta\cos\phi \\ \sqrt{2}R\sin\theta\sin\phi \end{pmatrix}\cdot R^2\sin\theta\begin{pmatrix} 2\cos\theta \\ \sin\theta\cos\phi \\ \sin\theta\sin\phi \end{pmatrix}d\theta d\phi$$
$$=\frac{1}{4\pi}\int\sin\theta d\theta d\phi=1,$$

which indicates that the singular point acts as the source of emergent magnetic field ($\nabla\cdot\mathbf{b}\neq 0$) and its effective charge is quantized.

Supplementary Note 2 │Comparison with magnetoresistivity in other isostructural helical magnets.

We compare magnetoresistivity (MR) profile in MnGe with those in isostructural helimagnets of MnSi and $Mn_{0.7}Fe_{0.3}Ge$ without emergent monopoles. In those helimagnets, we do not observe any peculiar MR profile, i.e., up-and-down H-dependence observed in MnGe (Fig. 2b in the main text). The absence of the distinct anomaly in other related helimagnets further emphasizes the positive MR anomaly as assigned to the fluctuations of emergent magnetic field. In particular, the comparisons exclude some other possible mechanisms of magnetic-field effect and spin-dependent scattering of electrons, as detailed below.

First, we examine the magnetic field effect on MR in the helical magnet of a single-crystalline MnSi with higher carrier mobility μ than that of the polycrystalline MnGe. Magnetic field gives rise to positive MR; for example, transverse MR in the single-carrier model obeys $\rho(B) = \rho_0[1 + (\mu B)^2]$, where a large positive MR is expected in the system with large mobility. This positive contribution may compete with the negative MR due to the reduction of scattering associated with the alignment of magnetization $\rho(M) \approx \rho_0 - a(M/M_s)^2$, which we described as the conventional negative MR in the main text; resulting in the peculiar up-and-down H-dependence. As reported by Lee *et al.*[2], the possible positive MR is hidden by the presence of dominant contribution from the negative MR nearly proportional to M^2. The absence of the distinct deviation from the conventional negative MR in MnSi even with higher mobility and less spin-charge coupling rules out the possibility that the magnetic-field-induced electron's cyclotron motion is the origin of the peculiar positive MR in MnGe.

Next, we also examine the possibility that spin-dependent scattering of electrons is the origin of non-monotonous H-dependence of MR. There is a theoretical report on the MR

effect in antiferromagnetic state[3], which shows similar H-dependence to those in MnGe, i.e., peak structure around the ferromagnetic transition. This profile is explained by the spin-fluctuation effect enhanced around T_N (antiferromagnetic transition temperature), which is effectively lowered by the magnetic field. Magnetic period of MnGe is very short (~3-6 nm), and hence the situation is close to the antiferromagnetic state; the spin-fluctuation effect possibly results in the positive peak in MR around H_c in MnGe. However, if this were the case, the helimagnet with very short magnetic period would show the similar MR profile. Here we compare MR in $Mn_{0.7}Fe_{0.3}Ge$ (Supplementary Figure 1), where short-period helical and skyrmion structures (~ 5 nm, comparable to that of MnGe) are observed by Lorentz transmission electron microscopy[4]. MR in $Mn_{0.7}Fe_{0.3}Ge$ clearly obeys the conventional negative MR almost proportional to M^2. Thus, the fact of short-period of magnetic structure is irrelevant to the positive MR due to the spin-fluctuation effect.

Supplementary Note 3 │Estimation of unusual contribution to positive magnetoresistivity.

We show procedures for the estimation of the unusual contribution to positive magnetoresistivity (MR), which we attribute to the fluctuations of emergent magnetic field. Supplementary Figure 2 shows longitudinal and transverse MRs as functions of normalized magnetization M/M_s at various temperatures. Here M_s is a saturated magnetization defined as the value at $T = 2$ K and $\mu_0 H = 14$ T. In both magnetic field directions, MR converges to trace an identical curve with elevation of temperature. We adopt MR at 200 K just above the transition temperature $T_N \approx 170$ K as the usual contribution to negative MR originating from the suppression of spin-flipping scattering due to magnetization alignment. We fit MR at 200 K to the following relation: $\rho(M) = \rho_0 - a(M/M_s)^2 - b(M/M_s)^4$, where ρ_0 is resistivity at zero field, a and b are coefficients for even terms under time-reversal operation.

The fitting curve is presented as black curves in Supplementary Figure 2. The unusual contributions to positive MR shown in Figs. 2e and 3b of the main text are determined as the difference between the measured MR (colour dots in Supplementary Figure 2) and the estimated usual negative MR (black curves in Supplementary Figure 2). The estimated unusual parts of longitudinal and transverse MRs are again presented in Supplementary Figure 2c,d. Here we note that the dip structure due to the suppression of emergent field fluctuation is faintly observed in longitudinal MRs at 20 K and 30 K, as indicated by closed triangles in Supplementary Figure 2c.

Supplementary Note 4 │Multi-peak-and-trench elastic profile with sound propagation direction perpendicular to magnetic field.

Sound wave-induced strains can modify the strengths of magnetic interactions in the material, equivalently yielding magnetoelastic interactions (see Methods). This leads to rich patterns of mixing up the three-mode magnon spectrum and the linear acoustic phonon spectrum, which are expressed as following relations and shown in Supplementary Figure 3a.

1st magnon linear mode: $\omega_1 = 2\hbar a_0 D k$

2nd magnon mode: $\omega_2 = 4eSb\frac{D^2}{J}\left(\sqrt{1+\left(\frac{\hbar a_0}{2eSb}\frac{J}{D}\right)^2 k^2} - 1\right)$, giving rise to a quadratic mode

$$\omega_2 = \frac{\hbar^2 a_0^2}{2eSb} J k^2 \quad \text{at small momentum}$$

3rd magnon mode: $\omega_3 = 4eSb\frac{D^2}{J}\left(\sqrt{1+\left(\frac{\hbar a_0}{2eSb}\frac{J}{D}\right)^2 k^2} + 1\right)$, showing a gap $\Delta = 8eSb\frac{D^2}{J}$

Phonon linear mode: $\omega_{\mathrm{ph}} = v_0 k$, where v_0 is the sound velocity.

Here we plot the spectra in Supplementary Figure 3a with parameters $a_0 = 2\pi/10$, $D = 2$, $J = 20$, $S = 1$, and $b = 0.1$ and with units $\hbar = e = v_0 = 1$ and magnetic period $= 2\pi$.

The drastic softening for $\mathbf{H} \parallel \mathbf{k}$ comes from a magnetic field-dependent hybridization between the two linear magnon and phonon excitations, which is maximized near the topological phase transition due to the immense contribution from spin-wave fluctuations. An intricate magnetic field-dependent evolution of the mixed effective phonon spectrum takes place in $\mathbf{H} \perp \mathbf{k}$ case because of the extra participation of the quadratic magnon mode, in addition to contribution from a similar linear-mode. Since the former quadratic mode is uniquely generated by the emergent magnetic field and the magnon stiffness is inversely proportional to b, the hybridization with phonon mode drastically varies upon increasing magnetic field until the topological phase transition. This complex mixing gives rise to multiple elastic anomalies even in a single magnetic phase in stark contrast to that in MnSi. For instance, the aforementioned extremal behaviour also finds itself in producing the left deepest trench.

In particular, we obtained the excellent consistency for the number and arrangement of the multiple peaks and trenches in the profile of the elastic constant with $\mathbf{H} \perp \mathbf{k}$. Supplementary Figure 3b,c shows typical theoretical and experimental results on the elastic constant with $\mathbf{H} \perp \mathbf{k}$, which have good agreement with each other. Multiple peaks and trenches are also observed in a wide temperature region below 110 K (Supplementary Figure 3d).

Supplementary Note 5 │Detail development of emergent magnetic field against magnetic field.

We describe in detail the external magnetic field dependence of the monopole positions and the emergent magnetic field.

Although the numbers of monopoles and antimonopoles in MnGe remain constant until the pair annihilation is caused by a high external magnetic field, i.e., the total topological charge in the system is always zero, the averaged emergent magnetic field continuously changes with a variation of the external magnetic field as shown in Fig. 2d of the main text. This profile contrasts with the magnetic skyrmion system[5,6], where its emergent field discretely changes with the number of skyrmions, leading to the sharp stepwise profile at the first-order transitions. By illustrating the monopole positions and the skyrmion number of every z-plane, we explain the continuous profile of the emergent field, which is the key for the critical phenomena as described in the main text.

As the averaged emergent field, which appears as topological Hall effect, is proportional to the Berry phase $\phi_z = \int_{\text{unit cell}} \mathbf{n}(\mathbf{r}) \cdot [\partial_x \mathbf{n}(\mathbf{r}) \times \partial_y \mathbf{n}(\mathbf{r})] dV$ in the magnetic unit cell, here we introduce the skyrmion number for every z-plane $N_{\text{sk}}(z) = \frac{1}{4\pi} \int \mathbf{n}(\mathbf{r}) \cdot [\partial_x \mathbf{n}(\mathbf{r}) \times \partial_y \mathbf{n}(\mathbf{r})] dxdy$, i.e., $\phi_z = 4\pi \int N_{\text{sk}}(z) dz$, to illustrate the spatial distribution of the Berry phase. Note that the skyrmion number $N_{\text{sk}}(z)$ for each z is quantized to be an integer, and monopole and antimonopole correspond to the discontinuous changes of $N_{\text{sk}}(z)$ by ± 1 as z crosses these points, i.e., $N_{\text{sk}}(z_0 + \delta z) - N_{\text{sk}}(z_0 - \delta z) = +1$ (monopole) and $N_{\text{sk}}(z_0 + \delta z) - N_{\text{sk}}(z_0 - \delta z) = -1$ (antimonopole), where z_0 is the z-coordinate of monopole or antimonopole.

In Supplementary Figure 4, we exemplify monopole positions and areas with the finite skyrmion number (-1) at typical magnetic fields (induced magnetizations m_z or

polarization factor n_z). At $m_z = 0$, pairs of monopoles and antimonopoles sit on the respective planes (blue planes in Supplementary Figure 4a) and the skyrmion number is always zero at every *z*-plane. Under application of magnetic fields (Supplementary Figure 4b-d), monopoles (yellow dots in Supplementary Figure 4) and antimonopoles (green dots in Supplementary Figure 4) move positive and negative *z*-directions, respectively, making gaps (gray shaded region in Supplementary Figure 4) between planes where monopoles and antimonopoles exist (yellow and green planes in Supplementary Figure 4, respectively). Incidentally, this means that finite magnetic monopole current flows along *z*-direction (magnetic field direction). The skyrmion number is -1 in the gaps, while it is 0 in the other regions. The gap with $N_{\mathrm{sk}} = -1$ grows to cover all the region at the colliding point ($m_z = 1, n_z = 0.457$), which corresponds to the maximum magnitude of topological Hall effect ($\phi_z = -\phi_0$) in Fig. 2g of the main text. When we further apply the magnetic field, monopoles and antimonopoles move negative and positive *z*-directions, respectively, to close the gaps and show pair-annihilation at $m_z = \sqrt{2}, n_z = 0.605$.

In summary, the continuous change in the emergent magnetic field is described by the volume fraction of the regions with the integer skyrmion number $N_{\mathrm{sk}} = -1$.

In a different perspective on the emergent magnetic monopoles, they can be regarded as end points of the skyrmion string. The skyrmion string carrying the gauge potential $N_{\mathrm{sk}} = -1$ acts as the fictitious solenoid in Aharonov-Bohm effect as in the case of the Dirac string connecting the Dirac monopoles, and attaches an additional Berry phase on the conduction electrons. The averaged emergent magnetic field is determined by the skyrmion string length along *z*-direction, which corresponds to the volume fraction of gray boxes in Supplementary Figure 4. The pair annihilation of emergent monopoles and antimonopoles can be also regarded as the annihilation of the skyrmion strings observed at $m_z = \sqrt{2}, n_z = 0.605$. (The pair annihilation does not occur at the collision of monopoles and antimonopoles not connected by the skyrmion string at $m_z = 1, n_z = 0.457$.)

Supplementary Note 6 | Magnetic-field dependence of fluctuation of emergent magnetic field.

The scattering of electrons from magnons enters through the quantum fluctuations of the emergent magnetic field (see Methods) and can then be captured by the correlation functions $\langle b_k, b_k \rangle$ $(k = x, y, z)$. We show the magnetic-field (polarization factor n_z) dependence of the correlation functions normalized by their values at $n_z = 0$ in Supplementary Figure 5. As we already described in the main text, the correlation functions exhibit an anisotropy $\langle b_x, b_x \rangle = \langle b_y, b_y \rangle > \langle b_z, b_z \rangle$ and get larger till an upsurge near the phase transition except a downward regime around the extremum of ϕ_z. The second-order phase transition of pair annihilation entails large fluctuations due to the drastic structural change in topology. And the fluctuation effect of the spin texture around the monopole collision should be relatively suppressed since neither a slight increase nor decrease in the magnetic field can notably alter the extremal texture. This profile thus illustrates the characteristics listed in the main text, i.e., the prior small hump as a result of the dip among the overall upward trend in scattering. Lastly, an electric current of MR effect is dominantly affected by the perpendicular emergent magnetic fields in the sense of Lorentz force, i.e., $\langle b_x, b_x \rangle$, $\langle b_y, b_y \rangle$ for longitudinal MR and $\langle b_y, b_y \rangle$, $\langle b_z, b_z \rangle$ for transverse MR. Therefore, the parallel current subject to stronger scattering gains larger MR.

Supplementary References

1. Park, J. H. & Han, J. H. Zero-temperature phases for chiral magnets in three dimensions. *Phys. Rev. B* **83,** 184406 (2011).

2. Lee, M., Onose, Y., Tokura, Y. & Ong, N. P. Hidden constant in the anomalous Hall effect of high-purity magnet MnSi. *Phys. Rev. B* **75,** 172403 (2007).

3. Usami, K. Magnetoresistance in Antiferromagnetic Metals. *J. Phys. Soc. Jpn.* **45,** 466–475 (1978).

4. Shibata, K. *et al.* Towards control of the size and helicity of skyrmions in helimagnetic alloys by spin-orbit coupling. *Nature Nanotech.* **8,** 723–728 (2013).

5. Lee, M., Kang, W., Onose, Y., Tokura, Y. & Ong, N. P. Unusual Hall Effect Anomaly in MnSi under Pressure. *Phys. Rev. Lett.* **102,** 186601 (2009).

6. Neubauer, A. *et al.* Topological Hall Effect in the *A* Phase of MnSi. *Phys. Rev. Lett.* **102,** 186602 (2009).
